# Supplementary material for: Identification and functional analysis of non-coding regulatory small RNA FenSr3 in Bacillus amyloliquefaciens LPB-18
Source: PeerJ. 2023 May 15;11:e15236. doi: 10.7717/peerj.15236 (PMC10194069; doi:10.7717/peerj.15236)
Supplement: Supplemental Information 4 [file peerj-11-15236-s004.zip › KO/CK-vs-T1_map/map04926.html]

KEGG PATHWAY: Relaxin signaling pathway - Reference pathway


|  |  |
| --- | --- |
| **Relaxin signaling pathway - Reference pathway** |  |

[
Pathway menu
| Organism menu
| Pathway entry
| Show description
| User data mapping
]

Human relaxin-2 ("relaxin"), originally identified as a peptidic hormone of pregnancy, is now known to exert a range of pleiotropic effects including vasodilatory, anti-fibrotic and angiogenic effects in both males and females. It belongs to the so-called relaxin peptide family which includes the insulin-like peptides INSL3 and INSL5, and relaxin-3 (H3) as well as relaxin. INSL3 has clearly defined specialist roles in male and female reproduction, relaxin-3 is primarily a neuropeptide involved in stress and metabolic control, and INSL5 is widely distributed particularly in the gastrointestinal tract. These members of relaxin peptide family exert such effects binding to different kinds of receptors, classified as relaxin family peptide (RXFP) receptors: RXFP1, RXFP2, RXFP3, and RXFP4. These G protein-coupled receptors predominantly bind relaxin, INSL3, relaxin-3, and INSL-5, respectively. RXFP1 activates a wide spectrum of signaling pathways to generate second messengers that include cAMP and nitric oxide, whereas RXFP2 activates a subset of these pathways. Both RXFP3 and RXFP4 inhibit cAMP production, and RXFP3 activate MAP kinases.

|  |  |  |
| --- | --- | --- |
| Reference pathway | 184% 150% 122% 100% 82% 67% 55% | 图片下载 |
